# Peer Assessment of Student Presentations: Key Takeaways and Lessons Learned

**DOI:** 10.7759/cureus.59809

**Published:** 2024-05-07

**Authors:** Gitanjali Khorwal, Raviprakash Meshram, Vikas Vaibhav, Rahul Sharma, Brijendra Singh, Salu Chandran, Kshitiza Sharma

**Affiliations:** 1 Anatomy, All India Institute of Medical Sciences, Rishikesh, Rishikesh, IND; 2 Forensic Medicine and Toxicology, All India Institute of Medical Sciences, Rishikesh, Rishikesh, IND; 3 Mental Health Nursing, All India Institute of Medical Sciences, Patna, Patna, IND

**Keywords:** feedback, pedagogy, rubric, assessment, anatomy, students’ presentation

## Abstract

Background

Students’ presentations are a teaching-learning tool where students not only study and understand a topic but also teach their peers, thereby learning the art and skill of effective presentation.

Aims

The study aimed to evaluate peer assessments in students’ presentations and find their role and application in improving presentation skills among students through feedback and course correction.

Methods

A group of students every week from a class of 125 was assigned a topic to present to the rest of their batch students who evaluated their presentation on a rubric shared via a Google Form link. The number of students who gave responses was noted. The responses were also shared with the presenters. The evaluator faculty moderated and discussed the areas for possible improvement and course correction. The students also filled out a feedback form on the entire exercise after presentations from the entire batch.

Results

The quality of students’ presentation skills improved with subsequent students over the period. The students learned about their areas of improvement. Through the feedback form, students shared their reasons not to give a presentation. However, most of them found the exercise beneficial.

Conclusions

Peer assessment can be a credible mode for improving presentation skills with the active participation of other students and provide a learning method based on others’ performances. Peer responses provide for self-evaluation and self-reflection. This type of survey among different institutions will identify students' flaws, help them improve and self-evaluate, and add to the current literature.

## Introduction

Didactic lectures are disparaged worldwide as an ineffective teaching method. They are monotonous, boring, and ineffective at holding students’ attention [1]. A large amount of information in the medical curriculum and limited interaction time induce nonparticipation among students [2].

Regardless, the increasing number of students and limited resources leave the faculty and instructors with few choices, and traditional didactic lectures still affect teaching [3]. This keeps educators from looking for methods to keep students attentive and interested in lectures [4]. Especially in basic sciences, subjects such as anatomy and pathology carry a high cognitive load because of the high volume of information; therefore, memorizing remains the keystone strategy [5]. Interactive approaches to increase active involvement by way of deeper learning and peer assessment during presentations could be a way to keep students interested and attentive [6].

Competency-based medical curriculum (CBME) by the National Medical Council (NMC) of India greatly emphasizes self-directed learning. One of the goals of an Indian Medical Graduate as a learner is to have effective communication skills with patients, families, colleagues, and the community [7].

Students’ presentations of topics that they prepare and present to the class are an efficient way to assess knowledge, skills, and attitude, while revising important topics, especially before the exams with the entire class. While this benefits the presenting students by allowing them to study the topic in depth, it also helps others by providing a quick revision. During the process, the students work on their performance styles and abilities to give an effective presentation [8]. This is particularly significant to inculcate a learner-centered approach among students necessary for better understanding and retention of an enormous number of facts and information in preclinical subjects [9].

With exact moderation and inputs from the evaluator faculty, students are objectively able to analyze the areas where they need to refine mentor outright correction. Here, in addition to evaluation by a faculty, we also arranged for a peer assessment of such presentations. Peer assessment in this system aimed to provide feedback from classmates in a nonthreatening manner to promote the development of professional communication soft skills. This resulted in self-evaluation, self-reflection, and overall enhancement in presentation skills.

## Materials and methods

Setting

The descriptive cross-sectional survey was conducted in 2021 in the Department of Anatomy. Students' feedback on novel peer assessment methods to improve presentation skills among students in the first year of their Bachelor of Medicine and Bachelor of Surgery (MBBS). The 125 enrolled participants were first-year students of MBBS in a Government-run medical college, out of which 86 (68%) were male and 39 (31.2%) were female students. Those who presented comprised 42 (33%) male and 19 (15%) female students. Non-presenters comprised 44 (35%) males, and 20 (16%) females. Students’ presentations are a part of routine teaching-learning methods. However, for this study, a web-based evaluation tool in the form of an Assessment Rubric [10] was included after the necessary permissions from the author, Michael J Peeters, via mail, Institutional Ethical Committee, and Dean [Academics]. The first author in this publication was the principal faculty in the Department of Anatomy and oversaw scheduling and conducting presentations. She was also responsible for disseminating emails, noting responses through assessment forms, and sharing them with presenters.

Stage 1: Conducting students’ presentations and receiving peer assessments

Students’ presentations were scheduled once a week, where a team of four students presented their topics through audio-visual mode. The session typically lasted for 50 minutes. Topics were assigned to students at least one week before the presentation. The related rubric (Appendix A, Table 4) for assessment of the presentation was mailed to the rest of the students as a Google Form. They were asked to give their responses on the presenter student’s performance. Responses were turned off at the end of the day. The responses recorded anonymously were shared with the presenter. A total of 31 sessions were scheduled for 125 students. Based on whether the students presented or not, they were grouped as presenters and non-presenters.

Stage 2: Receiving feedback

At the end of all 31 sessions, the students were mailed another Google Form for their feedback on the whole exercise of conducting students’ presentations and their assessment process. Two separate Google Forms with a few overlapping questions were mailed to the two groups, namely, presenters and non-presenters. The questionnaire contained mainly close-ended questions with a single open-ended question at the end regarding feedback (Appendix B, Table 4). The responses were tabulated, coded according to themes, and analyzed using Microsoft Excel 2016.

Statistical analysis

Baseline characteristics are presented as a pie chart and line diagrams as appropriate. The responses to the questions in the presentation rubric are portrayed as percentages (%) to show the frequency of student responses, the (n) value shows the presenter group, and (N) represents non-presenter students, respectively. The response to the overall feedback rubric is described as percentages (%) or using column charts as appropriate.

## Results

**Figure 1:**
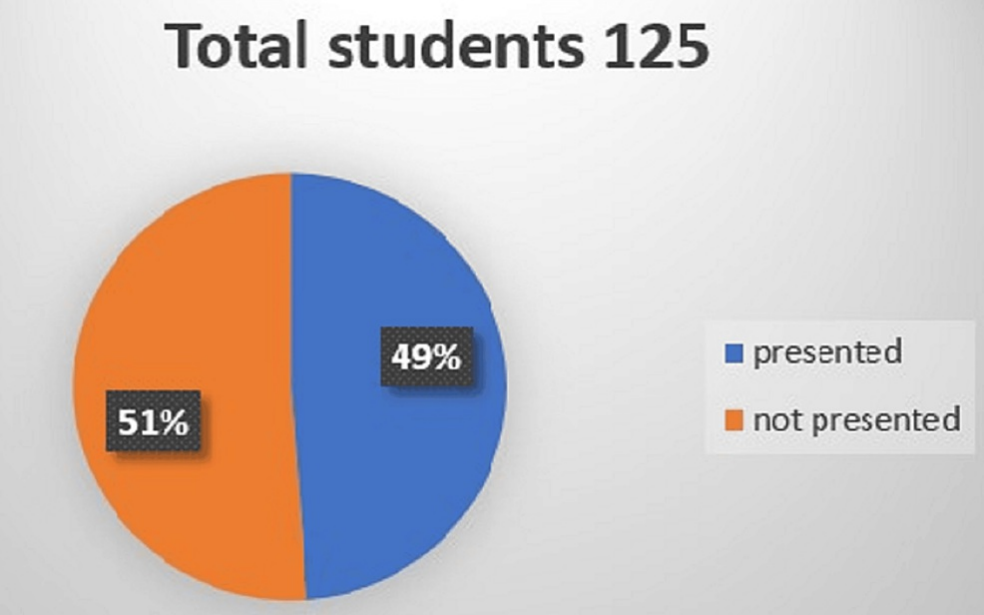
Proportion of students who presented their assigned topic. Total students N=125: presenters (N=61, 49%), non-presenters (N=64, 51%).

Out of the total 125 students in the batch, 61 (49%) presented their assigned topic in the class (Figure 1). Almost all students presented their topic using Microsoft PowerPoint. The reasons for not presenting were quoted as having stage fright, unpreparedness, and taking leaves because of festivities.

**Figure 2:**
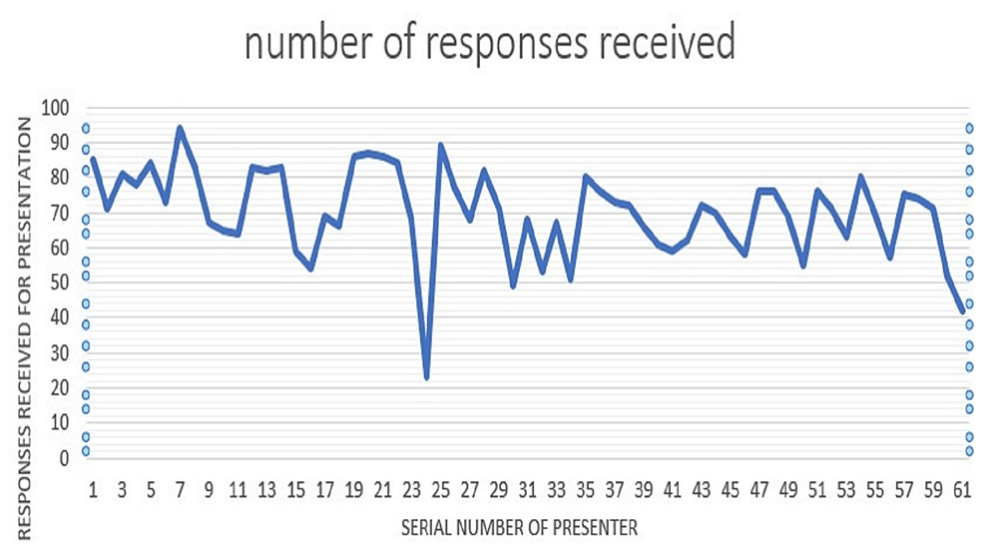
Number of responses given by audience students during the presentations. For instance, the first presenter received 85 responses for his/her presentation, the second received 71 responses, the third received 81 responses, and so on.

The number of responses given by audience students during the presentations (Figure 2). The number of responses varied in every session depending on the students that attended the session. Students either forgot to give responses during presentations, or their responses could not be submitted because of mobile phone network unavailability in classrooms.

Figures 3-13 show the responses recorded by students for every item of the rubric for assessing the student’s presentation. The same was shared with the students for self-evaluation and self-reflection.

**Figure 3:**
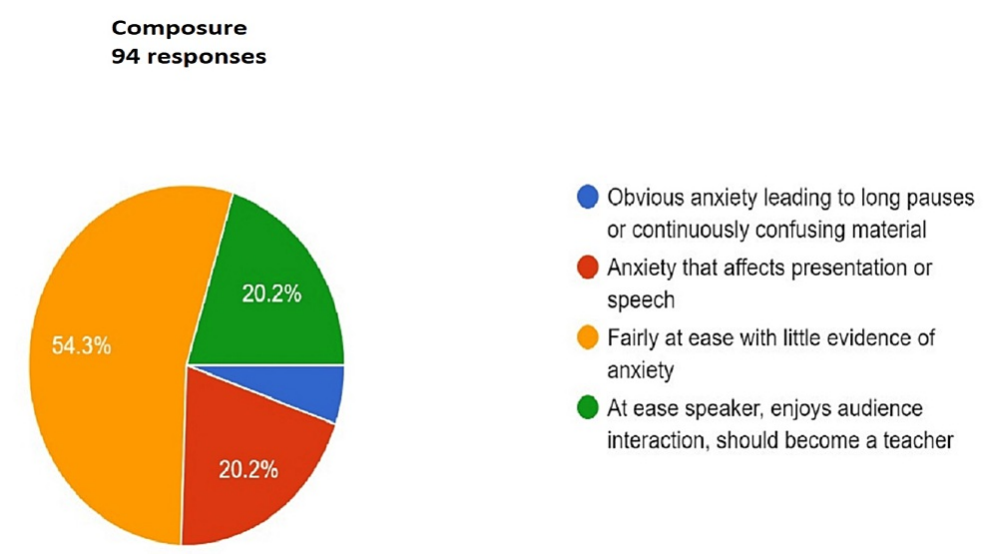
Assessment of composure. Total responses, N=94. In green, 20.2% (N=19) of the audience felt that the speaker was at ease, enjoys audience interaction, and should become a teacher; in yellow, 54.3% (N=51) of the audience felt the speaker was fairly at ease with little evidence of anxiety; in red, 20.2% (N=19) of the audience felt that the speaker has anxiety that affects presentation or speech; and in blue, 5.3% (N=5) of the audience felt that the speaker has obvious anxiety leading to long pauses or continuously confusing material.

**Figure 4:**
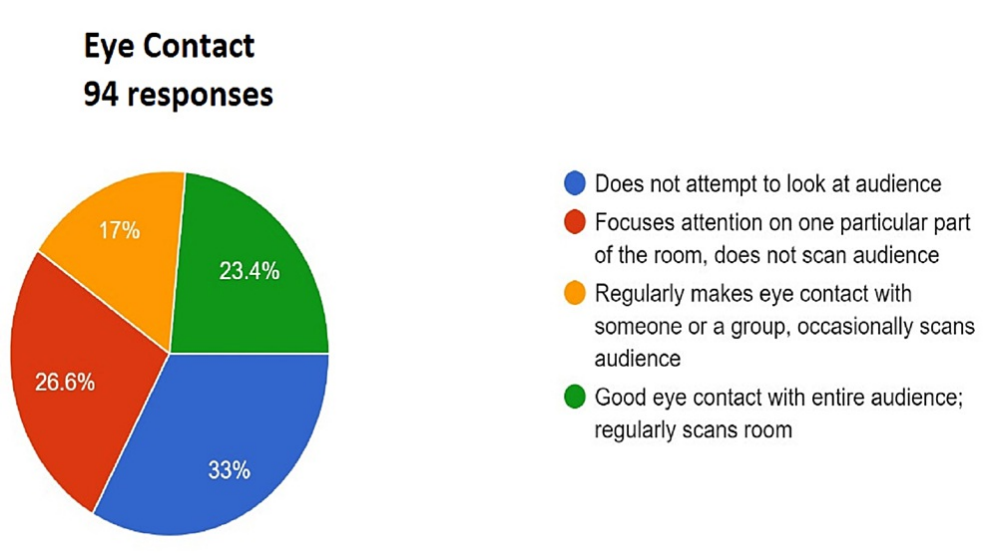
Assessment of enthusiasm/vocal pitch. Total responses, N=94. In green, 21.3% (N=20) of the audience felt that the speaker demonstrated a strong positive feeling about the topic during the entire presentation and used voice efficiently to emphasize points; in yellow, 41.5% (N=39) of the audience felt that the speaker generally shows positive feelings about the topic; some pitch variance; in red, 33% (N=31) of the audience felt that the speaker had absolute monotone; and in blue, 4.3% (N=4) of the audience felt that the speaker shows absolutely no interest in the topic presented or negativity toward the topic.

**Figure 5:**
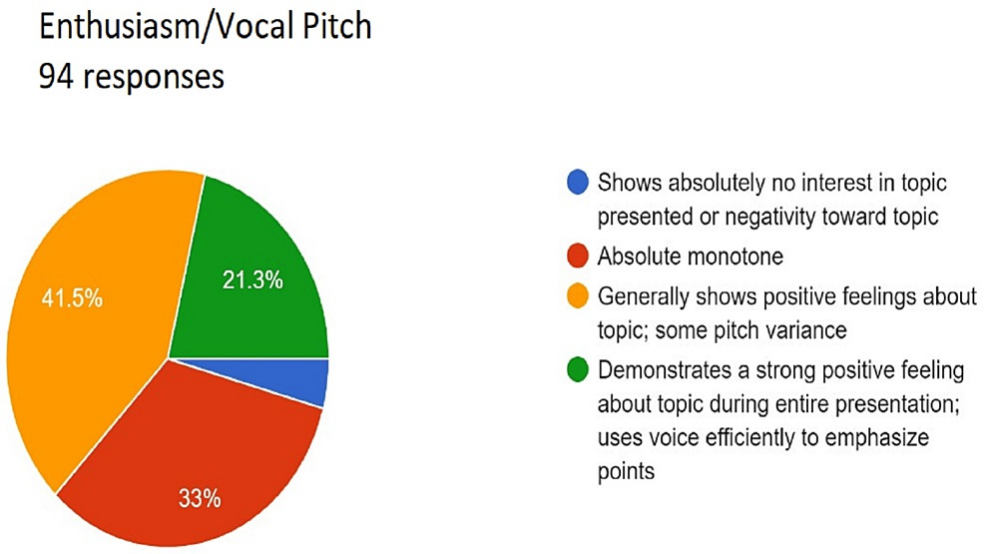
Assessment of enthusiasm/vocal pitch. Total responses, N=94. In green, 21.3% (N=20) of the audience felt that the speaker demonstrated a strong positive feeling about the topic during the entire presentation and used voice efficiently to emphasize points; in yellow, 41.5% (N=39) of the audience felt that the speaker generally shows positive feelings about the topic, some pitch variance; in red, 33% (N=31) of the audience felt that speaker had absolute monotone; and in blue, 4.3% (N=4) of the audience felt that speaker shows absolutely no interest in the topic presented or negativity toward the topic.

**Figure 6:**
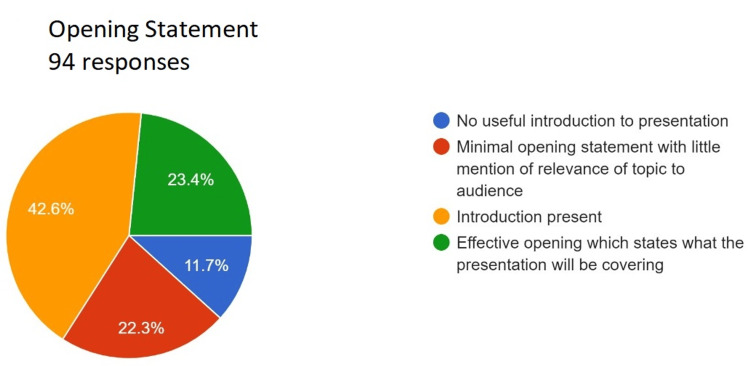
Assessment of opening statement. Total responses, N=94. In green, 23.4% (N=22) of the audience felt that the speaker gave an effective opening, which states what the presentation will be covering; in yellow, 42.6% (N=40) of the audience felt that the speaker gave the introduction; in red, 22.3% (N=21) of the audience felt that the speaker gave a minimal opening statement with little mention of the relevance of the topic to the audience; and in blue, 11.7% (N=11) of the audience felt that the speaker gave no useful introduction to the presentation.

**Figure 7:**
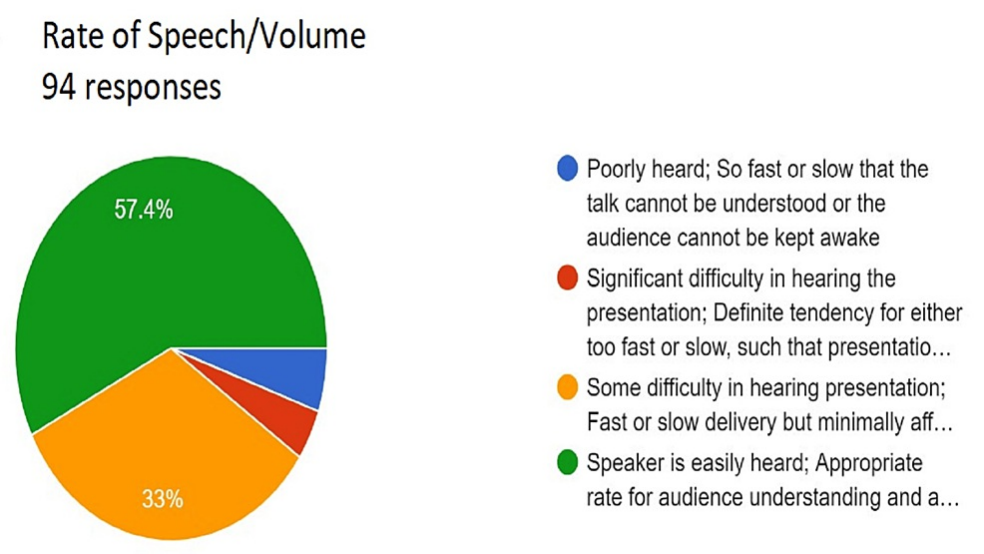
Assessment of rate of speech/volume. Total responses, N=94. In green, 57.4% (N=54) of the audience felt that the speaker is easily heard, appropriate rate for audience understanding and attention; in yellow, 33% (N=31) of the audience felt some difficulty in hearing the presentation and fast or slow delivery but minimally affects ability to follow presentation; in red, 4.3% (N=4) of the audience felt significant difficulty in hearing the presentation and definite tendency for either too fast or slow such that presentation is difficult to understand; and in blue, 5.3% (N=5) of the audience felt that the speaker was poorly heard and so fast or slow that the talk cannot be understood or the audience cannot be kept awake.

**Figure 8:**
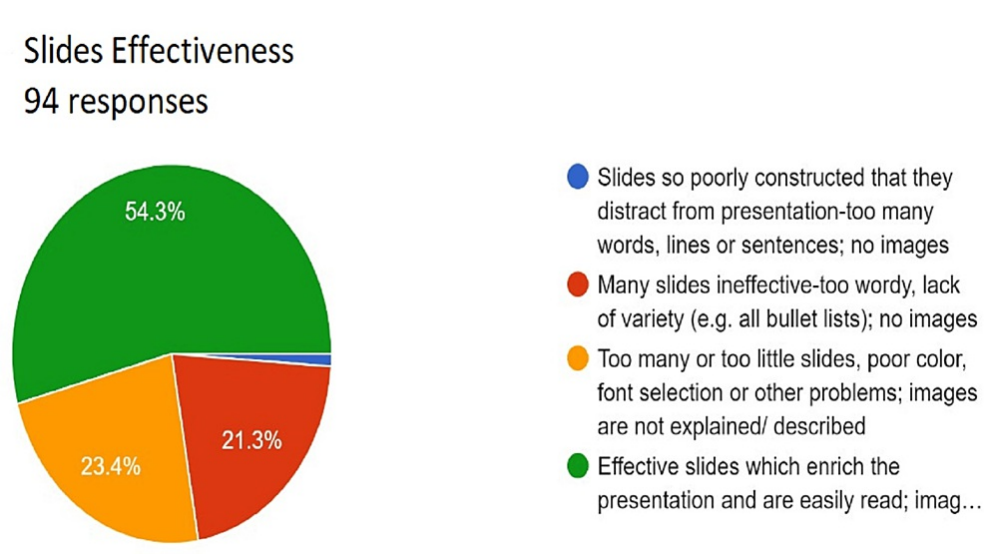
Assessment of slide effectiveness. Total responses, N=94. In green, 54.3% (N=51) of the audience felt that slides were effective, which enriched the presentation and were easily read and images used and described/explained throughout; in yellow, 23.4% (N=22) of the audience felt too many. In yellow, N or too few slides, poor color, font selection or other problems and images are not explained/described; in red, 21.3% (N=20) of the audience felt many; in yellow, N slides ineffective and too wordy, lack of variety (e.g., all bullet lists), no images; and in blue, 1.1% (N=1) of the audience felt slides were so poorly constructed that they distract from presentation-too many. In yellow, N words, lines or sentences, no images.

**Figure 9:**
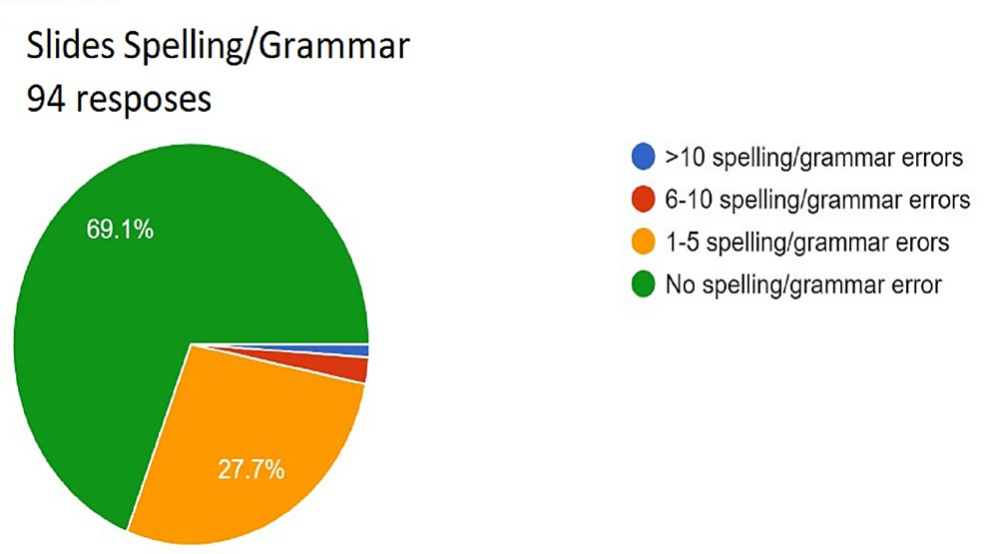
Assessment of slides spelling/grammar. Total responses, N=94. In green, 69.1% (N=65) of the audience found no spelling/grammar error); in yellow, 27.7% (N=26) of the audience found 1-5 spelling/grammar errors; in red, 2.1% (N=2) of the audience 6-10 spelling/grammar errors; and in blue, 1.1% (N=1) of the audience >10 spelling/grammar errors.

**Figure 10:**
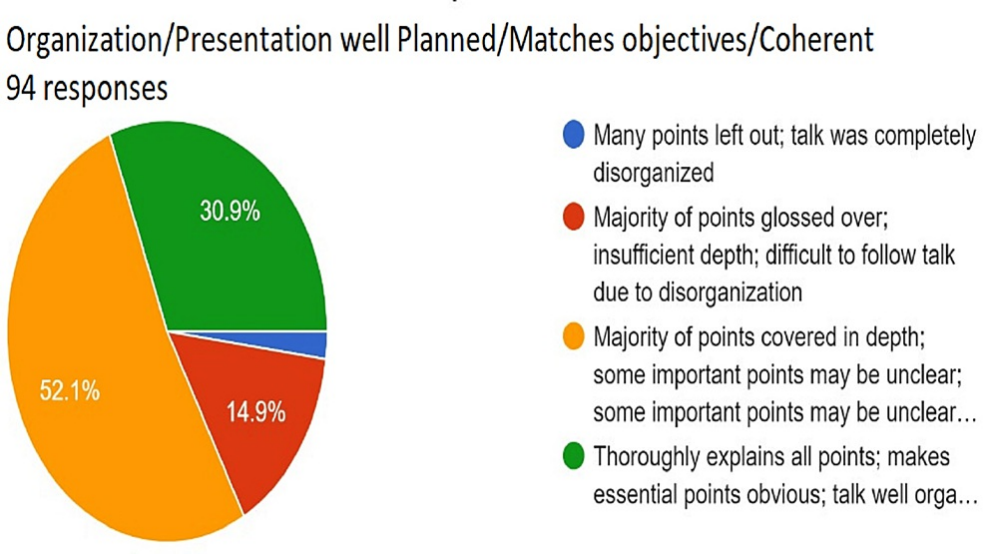
Assessment of organization/presentation well planned/matches objectives/coherence. Total responses, N=94. In green, 30.9% (N=29) of the audience felt that the speaker thoroughly explained all points and made essential points obvious and the talk was well-organized; in yellow, 52.1% (N=49) of the audience felt that the majority of points were covered in depth, some important points may be unclear, and some organization issues; in red, 14.9% (N=14) of the audience felt the majority of points glossed over, insufficient depth, and difficult to follow the talk because of disorganization; and in blue, 2.1% (N=2) of the audience felt that many. In yellow, N points were left out, and the talk was completely disorganized.

**Figure 11:**
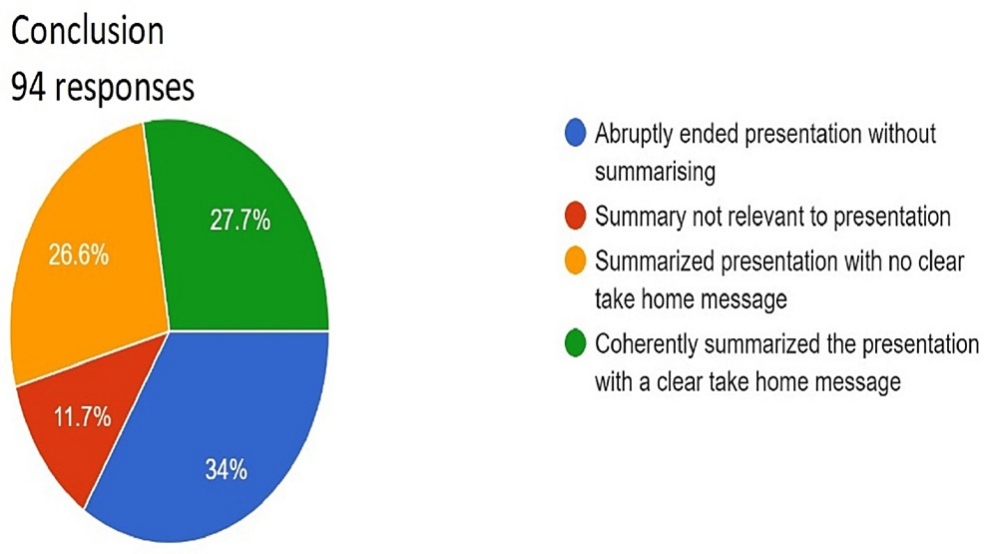
Assessment of conclusion. Total responses, N=94. In green, 27.7% (N=26) of the audience felt that the speaker coherently summarized the presentation with a clear take-home message; in yellow, 26.6% (N=25) of the audience felt that the speaker summarized the presentation with no clear take-home message; in red, 11.7% (N=11) of the audience felt summary not relevant to presentation; and in blue, 34% (N=32) of the audience felt that the speaker abruptly ended presentation without summarizing.

**Figure 12:**
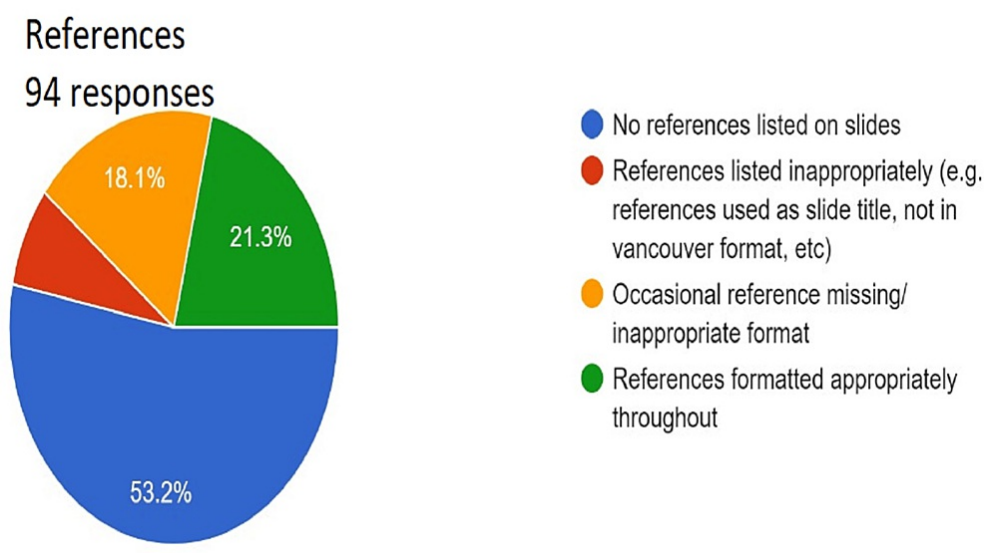
Assessment of references. Total responses, N=94. In green, 21.3% (N=20) of the audience opined that references were formatted appropriately throughout); in yellow, 18.1% (N=17) of the audience opined occasional reference missing/inappropriate format); in red, 7.4% (N=7) of the audience opined references listed inappropriately (e.g., references used as slide title, not in Vancouver format, and so on); and in blue, 53.2% (N=50) of the audience opined no references listed on slides.

**Figure 13:**
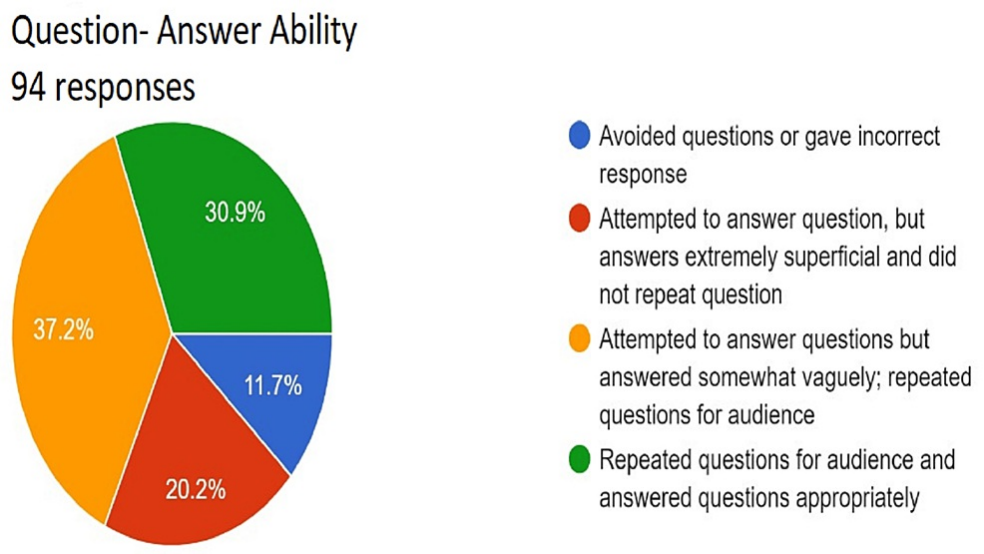
Assessment of question-answering ability. Total responses, N=94. In green, 30.9% (N=29) of the audience felt that the speaker repeated questions for audience and answered questions appropriately; in yellow, 37.2% (N=35) of the audience felt that the speaker attempted to answer questions but answered somewhat vaguely and repeated questions for audience); in red, 20.2% (N=19) of the audience felt that the speaker attempted to answer question, but answers extremely superficial and did not repeat questions; and in blue, 11.7% (N=11) of the audience felt that the speaker avoided questions or gave incorrect response.

**Figure 14:**
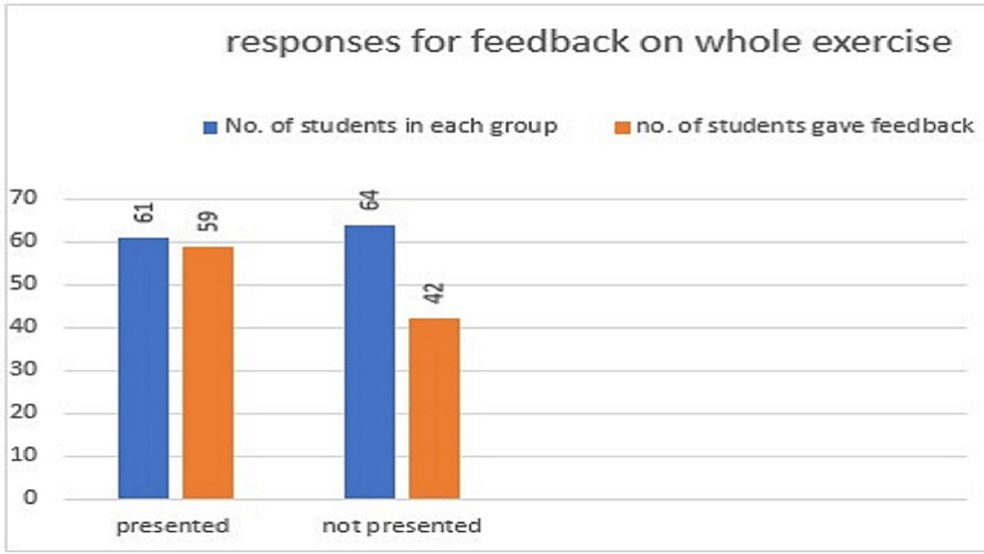
Figure showing responses for feedback on the whole exercise. Approximately 59 out of 61 (96.7%) students who gave a presentation also gave responses to the feedback form. Approximately 42 out of 64 (65.6%) students who did not give a presentation gave responses to the feedback form showing responses for feedback on the whole exercise.

Responses for the feedback questionnaire are given in Table 1 and Figure 14.

**Table 1:** Table showing responses for the feedback questionnaire.

Did you understand the purpose of the peer assessment in presentations?		
Response	Presenter group (N=61)	Non-presenter Group (N=64)
Yes	93.2% (N=55)	92.9% (N=39)
No	1.7% (N=1)	0
Maybe	5.1% (N=3)	7.1% (N=3)
Do you think the conduction of seminars was scheduled at the appropriate time during the first-year classes?		
Yes	96.6% (N=57)	95.2% (N=40)
No	3.4% (N=2)	2.4% (N=1)
Maybe	0	2.4% (N=1)
What would have been a more appropriate time to conduct such an exercise?		
Seminars were scheduled correctly (i.e., after finishing the syllabus)	72.9% (N=43)	73.8% (N=31)
Seminars should be scheduled once a week simultaneously with the ongoing classes	25.4% (N=15)	23.8% (N=10)
There is no need for seminars	1.7% (N=1)	2.4% (N=1)
Did attending seminars help you revise those topics easily?		
Yes	74.6% (N=45)	73.8% (N=31)
No	8.5% (N=5)	2.4% (N=1)
Maybe	16.9% (N=11)	23.8% (N=10)
Did attending other students' seminars help you understand areas to get better at your presentation skills?		
Yes	81.4% (N=49)	92.9% (N=39)
No	3.4% (N=2)	2.4% (N=1)
Maybe	15.3% (N=10)	4.8% (N=2)

Additional questions were asked to the presenter group in Table 2.

**Table 2:** Responses to additional questions asked to the presenter group.

Did preparing for seminars help you read and understand the topic better?	N=number of presenters
Yes	96.6% (N=58 )
No	0
Maybe	3.4% (N=2)
Did preparing for seminars help you improve your presentation skills?	
Yes	94.9% (N=56)
No	0
Maybe	5.1% (N=3)
Did you feel any increase in your confidence levels after the presentation?	
Yes	88.1% (N=52)
No	1.7% (N=1)
Maybe	10.2% (N=6)

Additional questions were asked to the non-presenter group as shown in Table 3.

**Table 3:** Responses to additional questions asked only to the non-presenter group.

Do you feel that by not presenting the seminar you lost an opportunity to improve your presentation skills?	N=number of non-presenters
Yes	88.1% (N=37)
No	4.8% (N=2)
Maybe	7.1% (N=3)
Do you still agree with your choice of not presenting as it was a difficult thing to do?	
Yes	19% (N=8)
No	61.9%
Maybe	19%

The responses from the presenter and non-presenter groups were largely similar. More than 70% of students said that seminars helped them revise the syllabus easily. More than 80% of students responded that attending other students' seminars helped them understand the areas to get better at their own presentation skills. Approximately 88% felt an increase in their confidence levels. A similar number of students, who did not present, felt that they lost a good opportunity to hone their presentation skills as it was not a very difficult thing to do (Table 3).

The qualitative data in response to the question “What is your overall opinion on such an exercise?” was tabulated. A total of 101 responses were collected. Overall, the students found the exercise to be good and helpful for revising the topics interestingly and efficiently, and preparation for seminars gave them a deeper understanding of the topics. It enhanced their presentation skills and boosted their confidence to present in front of a large audience. Moreover, it inculcated a sense of teamwork among them, and the responses shared with them helped in their self-evaluation. However, some students felt there was a scope for improvement in the execution of the exercise in terms of “flexibility” while assigning topics. An award or prize could further motivate students. However, frequent absenteeism among presenters led to the loss of certain topics, and students generally had a good experience of the exercise where both students and teachers were involved. They appreciated the inclusion of such an activity in the curriculum and wish to continue in the future.

For a presentation to be effective, engaging, and complete, it should cover a variety of aspects as mentioned in the Rubric (Appendix A) The responses collected from the peer audience were collated as a pie chart that gives a comprehensive visual presentation of the performance. The quality of the presentation is rated from worst to best (blue, red, yellow, and green in ascending order) in the pie chart. This readily gives the student visual feedback about areas where he or she performed up to the necessary standards and which areas require improvement. Sometimes, the presentation may be up to the mark in most areas but may lack a summary or clear take-home message. The responses by audiences encourage the presenter to look objectively at the aspects that need attention. Adding just one or two additional items can greatly enhance the overall quality of the presentation.

## Discussion

Healthcare professionals are frequently required to undertake presentations in a variety of settings. Classrooms, seminars, and panel discussions are a few venues to mention [11-13]. Despite the frequency and necessity to engage in such activities, fear of public speaking prevails in most people as they lack the skills [11-13]. Only 50% of students carried out their presentations owing to factors such as unpreparedness, stage fright, and so on.

The ability to present project work to students or fellow audience is nonnegotiable in a healthcare setting. However, this crucial skill is rarely taught in classrooms [14].

Although most people are interested in improving and enhancing their presentation skills, a survey conducted among 88 healthcare professionals states that “lack of training on best practices” is their largest impediment to success. Other factors included difficulty expressing in the English language, lack of technical skills, low confidence, and so on [11]. Exposing students in their medical education to a peer audience and receiving feedback early can prove an important stepping stone toward successful presentations later in their professional sphere.

Overall, the students found the exercise to be fruitful in helping them revise important syllabus topics before their end-term exams. Integrating peer teaching-learning activities for students within the academic timetable has favorable outcomes [15]. Those who presented stated an increment in confidence pertaining to their knowledge and skills. Moreover, it was observed that students were getting skillful in presentations toward the end of sessions. Presumably, they were able to reflect and be aware of the areas where they could avoid mistakes made by previous students. They were more organized in their content and likely used the rubric guidelines to enhance their presentation style. More than 80% of students felt that attending their peers’ seminars/presentations helped them to understand the areas where they needed improvement. Even the non-presenters thought it to be a good opportunity to practice and not a very difficult task to have missed. Having said that, peer teaching through presentation requires considerable time and effort. In the absence of any formal training, students learn by observing their own teachers [16]. Therefore, the role of the teacher cannot be limited to just giving information on a certain topic but also incorporating effective skills and techniques to keep it an interactive and more wholesome experience.

Notably, a higher number of students gave their responses among the presenter group (N=59/61, 96.7%) compared to the non-presenter group (N=42/64, 65.6%). Participation of students as presenters and respondents in feedback indicated enthusiasm among students who put effort into teaching and learning activities. Students who did not present were not interested in filling out a feedback form [17].

## Conclusions

Today’s students shall be tomorrow’s teachers. They must learn the necessary skills for effective teaching and communication to a wide audience and prepare for the role. The best way to learn is by teaching. An effective presentation is an amalgamation of presence, organization of thoughts and content, effective delivery, and interaction with the audience. Teaching their peers provides them an opportunity to enhance their learning, and they become more confident when they teach it to their peers. A deeper understanding of the topic develops their interest in studies.

Assessing peers keeps the students engaged in the teaching activity and learning at every step regarding knowledge and presentation skills. Assessment from peers provides non-threatening, friendly feedback, which serves as a template for self-reflection and self-improvement. Moreover, it provides direction for future research, as only half of the students gave their presentations at their turn, so remedial measures are required to increase students’ participation. Audience participation needs to be encouraged when assessing their peers. A qualitative study comprising feedback from each student regarding their self-reflection on assessment may have a definite impact on exercise. Conducting students’ seminars after finishing the syllabus for a session where students study a topic in-depth and present it to their peers is not only a beneficial way of revision but can also provide an opportunity to develop their presentation skills. Peer assessment of such presentations enhances active participation among students who provide their feedback to their presenter. This feedback is necessary for self-evaluation and self-reflection for presenters who objectively get to know about the areas where they lack and are likely to improve.

## Appendices

**Table 4:** Assessment rubric and feedback on peer presentations (Appendixes A and B).

Appendix A: Assessment Rubric
Assessment of roll no. XX. Hello dear student. Please provide feedback on your peer's presentation.
This exercise is a medical education project aimed at improving the presentation skills of students.
Click the circle that most closely corresponds to the student's performance in each respective category.
*Note: Please double-check the student's name before clicking on responses.
Composure
1. Obvious anxiety leading to long pauses or continuously confusing material
2. Anxiety that affects presentation or speech
3. Fairly at ease with little evidence of anxiety
4. Speaker at ease, enjoys audience interaction and should become a teacher
Eye Contact
1. Does not attempt to look at the audience
2. Focuses attention on one part of the room; does not scan the audience
3. Regularly makes eye contact with someone or a group; occasionally scans the audience
4. Good eye contact with the entire audience; regularly scans room
Enthusiasm/Vocal Pitch
1. Shows no interest in the topic presented or shows negativity toward the topic
2. Absolute monotone
3. Generally, shows positive feelings about the topic; some pitch variance
4. Demonstrates strong positive feelings about the topic during the entire presentation; uses voice efficiently to emphasize points
Opening Statement
1. No useful introduction to the presentation
2. Minimal opening statement with little mention of the relevance of the topic to an audience
3. Introduction present
4. Effective opening that states what the presentation will be covering
Rate of Speech/Volume
1. Poorly heard; so fast or slow that the talk cannot be understood, or the audience cannot be kept awake
2. Significant difficulty in hearing the presentation; definite tendency for either too fast or slow, such that the presentation is difficult to understand
3. Some difficulty in hearing presentation; fast or slow delivery but minimally affects the ability to follow the presentation
4. The speaker is easily heard; the appropriate rate for audience understanding and attention
Slides Effectiveness
1. Slides so poorly constructed that they distract from presentation: too many words, lines, or sentences; no images
2. Many slides ineffective: too wordy, lack of variety (e.g., all bullet lists); no images
3. Too many or too little slides, poor color, font selection, or other problems; images are not explained/described
4. Effective slides that enrich the presentation and are easily read; images used and described/explained throughout
Slides Spelling/Grammar
1. >10 spelling/grammar errors
2. 6-10 spelling/grammar errors
3. 1-5 spelling/grammar errors
4. No spelling/grammar error
Organization/Presentation Well Planned/Matches Objectives/Coherence
1. Many points were left out; the talk was completely disorganized
2. Majority of points glossed over; insufficient depth; difficult to follow talk because of disorganization
3. Majority of points are covered in depth; some important points may be unclear; some organizational issues
4. Thoroughly explains all points; makes essential points obvious; talks well-organized
Conclusion
1. Abruptly ended presentation without summarizing
2. Summary not relevant to presentation
3. Summarized presentation with no clear take-home message
4. Coherently summarize the presentation with a clear take-home message
References
1. No references listed on slides
2. References listed inappropriately (e.g., references used as slide title, not in Vancouver format, etc.)
3. Occasional reference missing/inappropriate format
4. References formatted appropriately throughout
Question Answering Ability
1. Avoided questions or gave an incorrect response
2. Attempted to answer questions, but answers were extremely superficial, and did not repeat questions
3. Attempted to answer questions but answered somewhat vaguely; repeated questions for the audience
4. Repeated questions for the audience and answered questions appropriately

Appendix B: Feedback on Peer Presentations
Thank you for your participation in the students' presentations. We would like to know your opinion.
Questions Asked to Both Presenter and Non-Presenter Groups
Did you understand the purpose of peer assessment in presentations?
1. Yes
2. No
3. Maybe
Do you think the conduction of seminars was scheduled at an appropriate time during the 1st year classes?
1. Yes
2. No
3. There is no need for seminars
What would have been a more appropriate time to conduct such an exercise?
1. Seminars were scheduled correctly (i.e., after finishing the syllabus)
2. Seminar should be scheduled once a week simultaneously with the ongoing classes
3. There is no need for seminars
Did attending seminars help you revise those topics easily?
1. Yes
2. No
3. Maybe
Did attending other students' seminars help you understand areas to get better at your own presentation skills?
1. Yes
2. No
3. Maybe
What is your overall opinion on such an exercise?
Questions Asked to Presenter Group Only
Did preparing for seminars help you read and understand the topic better?
1. Yes
2. No
3. Maybe
Did preparing for seminars help you improve your presentation skills?
1. Yes
2. No
3. Maybe
Did you feel any increase in your confidence levels after the presentation?
1. Yes
2. No
3. Maybe
Questions Asked to Non-Presenter Group Only
Do you feel that by not presenting the seminar you lost an opportunity to improve your presentation skills?
1. Yes
2. No
3. Maybe
Do you still agree with your choice of not presenting as it was a difficult thing to do?
1. Yes
2. No
3. Maybe
